# Quantitative biochemical phenotypic heterogeneity of senescent macrophage at a single cell level by Synchrotron Radiation Fourier Transform Infrared Microspectroscopy

**DOI:** 10.1007/s00604-023-05980-z

**Published:** 2023-09-28

**Authors:** Xiaolong Sheng, Jie Wu, Xun Wu, Lianghui Gong, Min Su, Jinming Tang, Desong Yang, Wenxiang Wang

**Affiliations:** 1 The Second Department of Thoracic Surgery, Hunan Cancer Hospital/the Affiliated Cancer Hospital of Xiangya School of Medicine, Central South University, Changsha, China; 2 Hunan Clinical Medical Research Center of Accurate Diagnosis and Treatment for Esophageal Carcinoma, Changsha, China

**Keywords:** Macrophage senescence, Age-related diseases, SR-FTIR microspectroscopy, Biochemical phenotype and phenotypic heterogeneity

## Abstract

Macrophage senescence plays an important role in pathophysiological process of age-related diseases such as atherosclerosis, chronic obstructive pulmonary disease (COPD), pulmonary fibrosis, and lung cancer. After macrophage senescence, the biochemical phenotypes related to biological functions showed great heterogeneity. However, the biochemical phenotype and phenotypic heterogeneity of senescent macrophage has not been fully understood. Exploring the phenotype of biochemical substances in senescent macrophage will be helpful for understanding the function of senescent macrophage and finding out the potential mechanism between immune macrophage senescence and age-related diseases. In this study, we employed SR-FTIR microspectroscopy to detect the biochemical phenotype and phenotypic heterogeneity of single macrophage. The whole infrared spectra of senescent macrophages shifted, indicating biochemical substance changes within senescent macrophages. PCA and intercellular Euclidean distance statistical analysis based on specific spectra regions revealed dynamic changes of lipids and proteins during macrophage senescence. This proved that SR-FTIR microspectroscopy is an effective tool to detect the single cell biochemical phenotype transformation and phenotypic heterogeneity during macrophage senescence. It is of great significance to provide an evaluation method or clue for the study of cellular functions related to intracellular biochemical substances.

**Graphical abstract:**

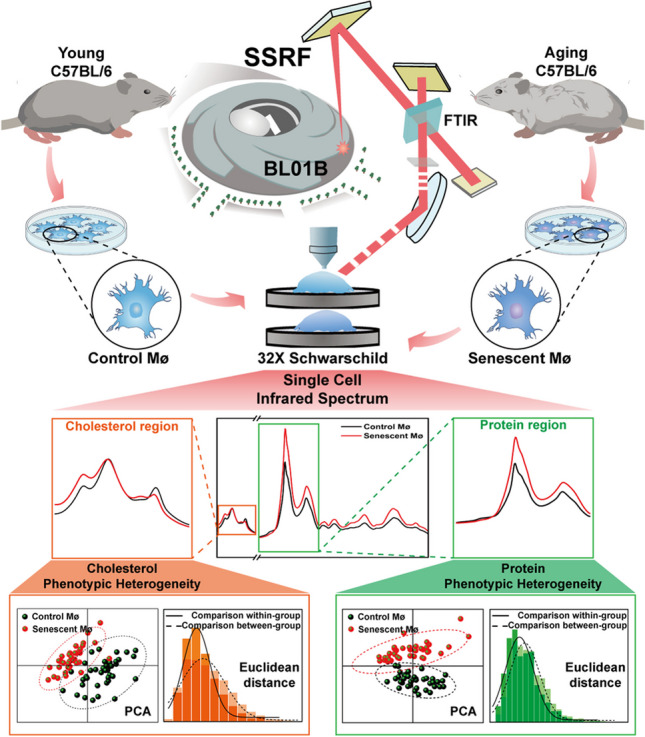

**Supplementary Information:**

The online version contains supplementary material available at 10.1007/s00604-023-05980-z.

## Introduction

Aging and cellular senescence are intricate biological phenomenon leading to numerous changes in the physiological systems [1–4]. Despite the biological effects of aging and senescence are relatively well understood, immune senescence, particularly macrophage senescence, is only beginning to be elucidated. It has been proved in several experimental findings that macrophage senescence plays an important role in atherosclerosis [5], chronic obstructive pulmonary disease (COPD) [6, 7], pulmonary fibrosis [8], lung cancer [9], and other age-related diseases [10, 11]. Once senescence occurs, macrophages undergo great heterogeneity in cell substance metabolism, phenotypic, function transformation, and biochemical components [12–15]. However, the biochemical phenotype and phenotypic heterogeneity of senescent macrophage has not been fully understood [16]. As the executor of cellular functions, intracellular macromolecular biochemical substances are closely related to cellular functions. Detecting the phenotypes of biochemical macromolecule including lipids, proteins, and carbohydrates in senescent macrophage at a single cell level is necessary and helpful for understanding the phenotypes and functions of macrophage senescence [17]. In fact, macrophage cellular populations in different physiological conditions turn on different molecular signals, acquire different biochemical phenotypes, and then display different functions [18]. It is difficult to analyze the exact changes of macrophage senescence by traditional methods [19]. Messenger Ribonucleic Acid detecting, Protein expression level quantify, SA-β-gal staining, immunofluorescence, and flow cytometry just have the limit ability to detect the main substance composition changes in senescent macrophages [20, 21]. These approaches also need a high-quality extraction process and chemical or fluorescent labeling for samples and could not obtain multiple biochemical component information changes. The population mean statistics can identify predominant compositions and interactions in cell population, but do not show differences between single cells. With the rapid development of science and technology, genomics, epigenomics, proteomics, and metabolomics which permit a comprehensive analysis of heterogeneity in a large cell population have been employed in unveiling cellular heterogeneity [22–24]. All these omics not only demand rigorous procedures of sample preparation but also intricate experiments or data processes.

Owing to several scientific researches, Synchrotron Radiation Fourier Transform Infrared (SR-FTIR) microspectroscopy has been developed into a powerful system for detecting biochemical information and spatial distributions with characteristics of nondestructive, high resolution, and label-free during biological analysis [25]. Through SR-FTIR, we could identify different chemical molecules or substances by analyzing the infrared absorption peaks of specific functional groups among the multi-dimensional wavelength [26]. Spectral diversity reflects small biochemical changes in the cell population, and single-cell SR-FTIR can be used to detect cell-to-cell variability, which is beyond the capacity of average cell spectral analysis [27]. In other words, SR-FTIR has the advantages of being more objective, accurate, and detailed to quantify the comprehensive molecular structures of cell heterogeneity, even at a single cell level synchronously [28]. It has been reported that SR-FTIR microspectroscopy can be used for investigating substance composition in tissues [29], detecting effects of stimuli on cells, tracking the biochemical characteristics during cell differentiation [30], and screening potential targets for disease drug therapy [31].

In this study, SR-FTIR was employed to detect the changes of biochemical macromolecular in senescent macrophage at a single cell level and further to reveal the biochemical phenotype and phenotypic heterogeneity. The results shown that proteins and lipids (especially cholesterol esters) took dominate changes during macrophage senescence. The study provides reliable spectroscopic evidence for the changes in biochemical components after macrophage senescence. It is of great value for understanding the deposited metabolites, biochemical phenotype, phenotypic heterogeneity, and functional diversity of senescent macrophage.

## Materials and methods

### Experimental animal

C57BL/6 mice (young: 2-month-old; aging: 18-month-old) were used for macrophages collection and maintained in pathogen-free animal facility. All mice have access to water and food with a 12 h cycle of light/dark in the laboratory Animal Unit. The whole experimental procedures were approved by the Animal Ethics Committee of Hunan Cancer Hospital/the Affiliated Cancer Hospital of Xiangya School of Medicine, Central South University.

### Isolation of alveolar macrophages

Alveolar macrophages were collected from 2-month-old (young) and 18-month-old (aging) C57BL/6 mice. All primary macrophages were isolated by alveolar lavage with ice-cold 1× PBS. Macrophages were seeded at a density of 3–5 × 10^6^ cells per well in DMEM (Gibco, Grand Island, NY, USA) with 10% fetal bovine serum (FBS, Biological Industries, Kibbutz Beit-Haemek, Israel) and 100 U ml^−1^ penicillin and streptomycin and maintained at 37 °C, 5% CO^2^. After overnight culture, washed cells with medium thrice and the adherent cells were alveolar macrophages.

### Immunofluorescence

Alveolar macrophages were fixed with 4% paraformaldehyde and washed with PBS thrice. Then permeabilized with 0.1% Triton X-100 (Cat No.:1139ML100, Biofroxx, Germany) and finally blocked with 3% BSA (Cat No.:143066, Biofroxx, Germany), Primary anti-F4/80 antibody (1:400; ab6640; Abcam, Cambridge, MA, USA) was added and incubated overnight at 4 °C. This was followed by incubation with the following secondary antibodies: donkey anti-rat Alexa Fluor 594 (Jackson Immunoresearch Laboratories, West Grove, PA, USA). Then stained nuclei with 4′, 6-diamidino-2-phenylindole (DAPI; 0.5 μg/mL; Invitrogen, Carlsbad, USA), and microimages were obtained using a fluorescence microscope (Carl Zeiss Axio Observer Z1, Oberkochen, Germany).

### SA-β-gal staining of macrophages

Alveolar macrophages, cultured in 24-well sterile culture plates, were fixed with 0.5-ml β-gal staining stationary solution for 15–30 min. Then incubated with 0.5–1-ml SA-β-gal staining working fluid (RG0039, Shanghai Beyotime Biotechnology Co., Ltd). Cells were then observed under an inverted microscope (Zeiss Scope, Zeiss, Heidenheim, Germany).

### Quantitative reverse transcription PCR (qRT–PCR)

Total RNA was extracted from sorted alveolar macrophages using Trizol (Invitrogen, Carlsbad, CA, USA) according to the manufacturer’s protocol. Each sample was run in triplicate for analysis. At the end of the PCR cycles, melting curve analysis was performed to validate the specific generation of the expected PCR product. The expression levels of mRNAs were normalized to GAPDH and were calculated using the 2−ΔΔCt method. Primer sequences for qRT–PCR were summarized in Table 1.

**Table 1:** Primer sequence for qRT–PCR

Gene	Forward (5′→3′)	Reverse (5′→3′)
Gapdh	ATGACATCAAGAAGGTGGTG	CATACCAGGAAATGAGCTTG
p16	CGTGAACATGTTGTTGAGGC	GCAGAAGAGCTGCTACGTGA
p21	CAGATCCACAGCGATATCCA	ACGGGACCGAAGAGACAAC
IL-1β	CCAAAAGATGAAGGGCTGCT	TCATCAGGACAGCCCAGGTC
IL-6	CTGCAAGAGACTTCCATCCAG	AGTGGTATAGACAGGTCTGTTGG
IL-10	CCTGGTAGAAGTGATGCCCC	TCCTTGATTTCTGGGCCATG
TNF-α	ATGCTGGGACAGTGACCTGG	CCTTGATGGTGGTGCATGAG
MMP12	GGGCTGCTCCCATGAATGAC	CCAGAGTTGAGTTGTCCAGTTG
MIF	GAGGGGTTTCTGTCGGAGC	GTTCGTGCCGCTAAAAGTCA

### Synchrotron Radiation Fourier Transform Infrared (SR-FTIR) micro spectroscopy

The macrophages were washed with PBS twice. Cells were detached by using a cell scraper and then collected with 1.5-ml centrifuge tubes. After centrifugation at 600 × g for 10 min at 4 °C and discard the supernatant. All cells were fixed with 4% paraformaldehyde for 30 min at room temperature. Re-suspended the fixed macrophage after washing with ultrapure water thrice. Ten- to fifteen-microliter cell suspension was dripped on one piece of BaF_2_ window plate and dry under indoor temperature condition completely until all single macrophage would spread on the BaF_2_ window plate. All of the macrophage samples were preserved at 4 °C before experiments.

Single macrophage SR-FTIR microspectroscopy measurements were conducted at BL01B beamline of Shanghai Synchrotron Radiation Facility (SSRF). Each spectrum contain wavenumber region (4000–600 cm^−1^) was collected under aperture size 20 × 20 μm, resolution of 4 cm^−1^ and 64 co-added scans condition. After collected background from blank area, single spectra of individual macrophage on the BaF_2_ window plate were collected one by one; a total of about forty single cells were collected for each group (young: control macrophage; aging: senescent macrophage).

### Pretreatment of spectral data

All SR-FTIR spectrum were smoothed (9-point) and corrected baseline in OMNIC (Thermo Fisher Scientific Inc, USA) automatically. The Mie scattering was corrected by applying the RMie-EMSC algorithm as Paul Bassan described [32, 33]. Then, the second derivative spectra were calculated on OMNIC.

### Multivariate analysis of infrared (IR) data

Besides the whole spectral region of every macrophage, the regions of lipids (3000–2800 cm^−1^ together with 1480–1300 cm^−1^), proteins (1800–1480 cm^−1^), and nucleic acids and carbohydrates regions (1300–900 cm^−1^) were selected, respectively, for multivariate analysis.

### Euclidean distance and cellular Euclidean distance matrices between macrophages

The similarity between macrophages contain with “*N*” dimensional infrared spectra respectively were assessed by Euclidean distance:

$$d\left(a,b\right)=\sqrt{\sum_{i=1}^N\limits{\left({a}_i-{b}_i\right)}^2}$$

where *a* and *b* were two single cell represented by “*N*” dimensional vectors (“*N*” represented the number of infrared spectra points). The value of *d* (*a*, *b*) is negative correlate to intercellular similarity. Euclidean distance matrix was constructed by calculating all the cell-to-cell distances among In-group (control macrophages) or Inter-group (control macrophages and senescent macrophages). One group contains with “*n*” macrophages, a total number of ${C}_n^2=n\left(n-1\right)/2$. Euclidean distances would be acquired. While there were two groups (contain with “*x*” and “*y*” macrophages respectively), we could acquire a total of *x* × *y* Euclidean distances.

### Principle component analysis (PCA) and hierarchical cluster analysis (HCA)

Based on the single macrophage preprocessed and corresponding second derivative spectra IR data among In-group or Inter-group, we carried out principal component analysis (PCA) and hierarchical cluster analysis (HCA). For PCA, the first two principal components were used to construct the score plots through Origin Pro 2021b (OriginLab Inc, USA). The corresponding loading plots of PC1 and PC2 were also acquired. For HCA, the cellular Euclidean distance matrices were imported into the “Heat Map of Dendrogram” App of Origin Pro and “Ward Link” cluster method and “Euclidean” distance type were selected to form the cluster tree which were then visualized as hierarchical clustering (HAC) heat maps.

## Results

**Fig. 1:**
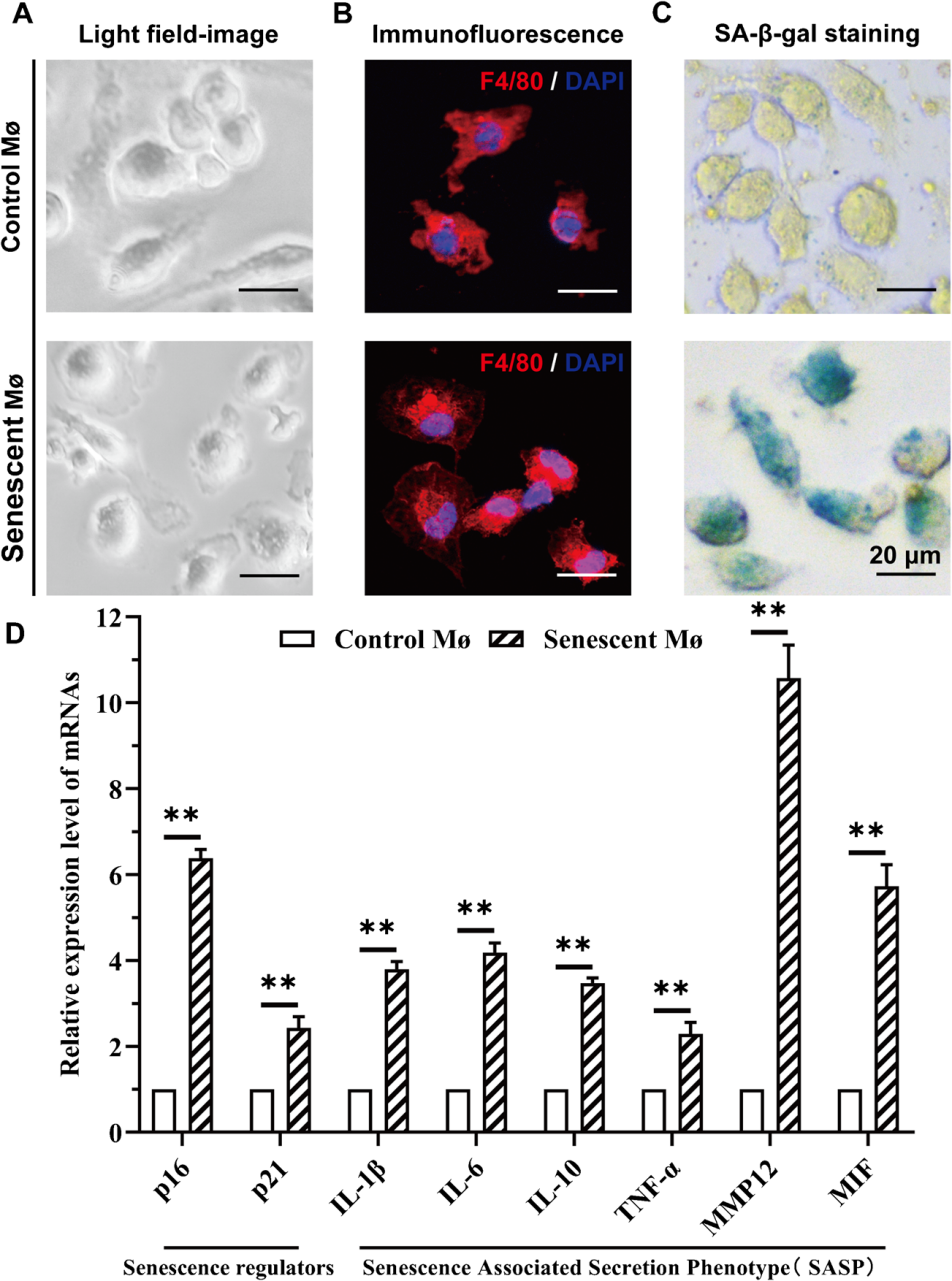
The identification of alveolar macrophages and senescent macrophages. **A** Light field-image of macrophages. **B** Immunofluorescence identification of macrophages (red: F4/80, blue: DAPI). **C** SA-β-gal staining of macrophages. Scale bar = 20 μm. **D** Relative mRNA expression level of aging-related factors

First of all, according to the experimental procedure described in materials and methods, we successfully isolated alveolar macrophages, then identified the senescence through SA-β-gal staining and qRT–PCR detection of aging-related factors (Fig. 1), and then collected additional alveolar macrophages for subsequent testing. Before Synchrotron Radiation Fourier Transform Infrared (SR-FTIR) experiments, thousands of macrophages (control macrophages from the young mice and senescent macrophages from the aging mice) were distributed on the barium fluoride (BaF_2_) window plate under a room temperature to dry completely.

### Infrared biochemical phenotype and phenotypic heterogeneity of macrophage senescence and the quantitative analysis based on the whole infrared spectra (3000–2800 cm^−1^ and 1800–900 cm^−1^)

As an immune cell possessed abnormal plasticity, macrophages shown extremely complex heterogeneity and functional phenotypes during senescence, and these phenotypical diversities were closely related to the changes of biochemical substance components within macrophages. The prepared macrophage samples were detected in sequence under the FTIR micro spectroscopy in the Synchrotron Radiation Facility at Shanghai. Ultimately we acquired a total of eighty (forty cells per group) high-quality infrared spectra for further analysis. In a typical SR-FTIR spectrum, the C-H bond stretching related to lipids was located in 3000–2800 cm^−1^ wave band and the spectral band near 1456 cm^−1^ was derived from CH_2_ bending vibrations. Fatty acid side chains COO^−^ symmetric stretching vibrations induce the band near 1390 cm^−1^regions. Bands located at near 1650 cm^−1^ and 1545 cm^−1^ are originate from Amide I and Amide II of proteins which caused by the combined contributions of N-H bending and C=O stretching; C-N stretching and N-H bending [33]. The band near 1084 cm^−1^ indicates the PO^2−^ phosphodiesters stretch of phosphorylated molecules and glycogen. The spectral absorption band near 984 cm^−1^ is on account of the C-O stretching from ribose nucleic acid (RNA) ribose chain [34, 35].

**Fig. 2:**
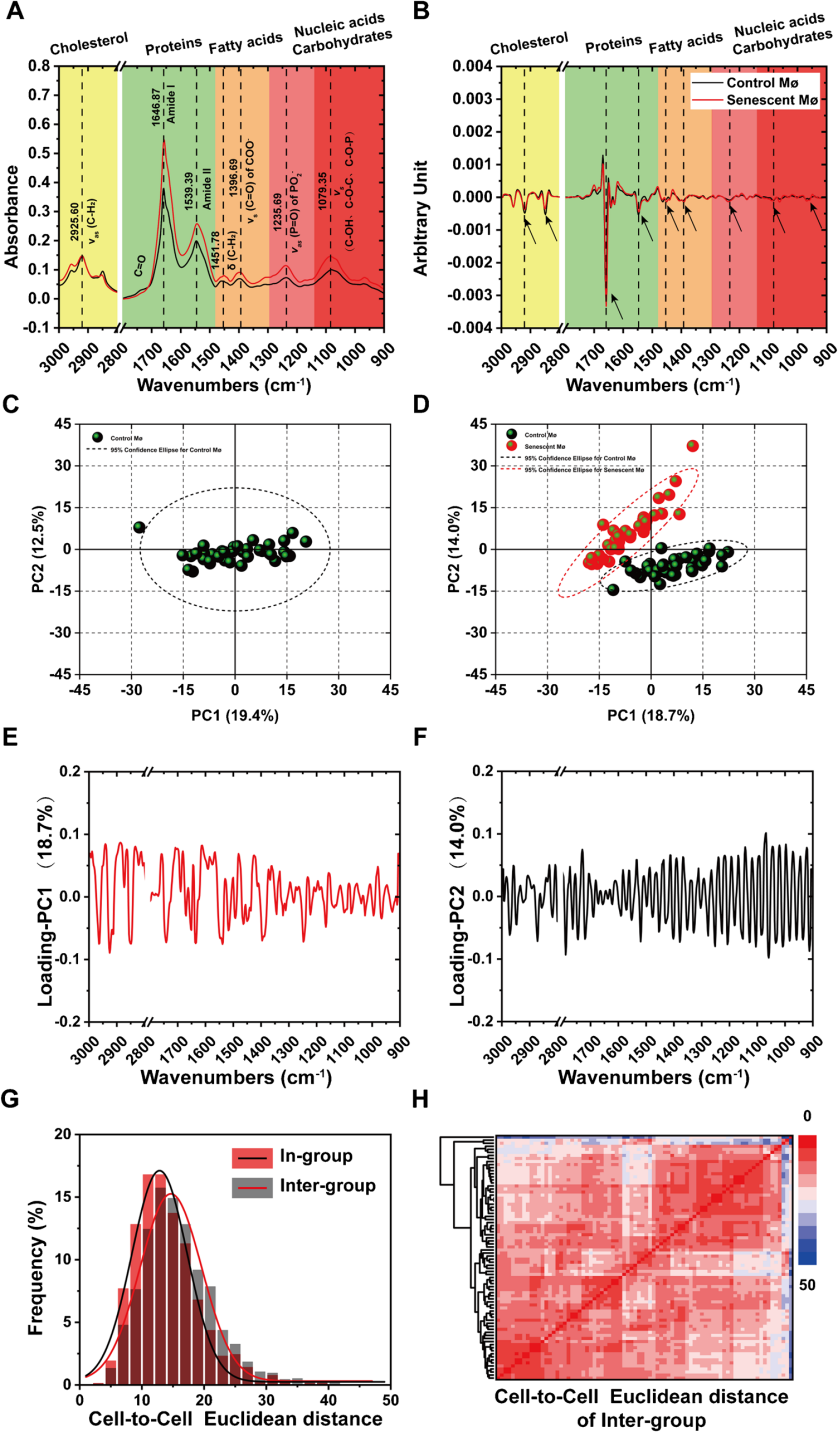
Infrared biochemical phenotype of macrophages and quantitative analysis of cellular heterogeneity. **A** The average SR-FTIR spectra shown the absorption spectral diversities in the band positions and intensities between the two groups, the corresponding second derivative spectra are shown in **(B)** (black line represents control macrophages of young group; red line represents senescent macrophages of aging group). **C** and **D** PCA of In-group (control macrophages) and Inter-group (control macrophages and senescent macrophages), the black dots represent samples of control macrophages and red dots represent samples of senescent macrophages; the ellipse circled by the dotted lines are the 95% confidence ellipse; control (black) and myelin debris-treated (red). **E** and **F** The corresponding loading plots of PC1 and PC2 of **(D)**. **G** Frequency distribution histograms indicate the distribution of cell-to-cell Euclidean distances of two cells population. The black line is fitted Gauss curves of In-group (control macrophages) and red line is fitted Gauss curves of Inter-group (control macrophages and senescent macrophages). **H** Hierarchical clustering (HAC) heatmap of cell-to-cell Euclidean distances show the biochemical phenotypic heterogeneity of these detected macrophage population in some infrared spectral absorption bands

After statistically analyzing the spectral datasets in the same batch, we successfully obtained the average infrared spectral absorption bands of young group and aging group (Fig. 2A and B). Around different particular functional group, the results display dramatic variations between the average infrared spectra and the corresponding second derivative spectra in the position and intensity of the absorption bands clearly. Then, we hypothesized that there was significant biochemical phenotypic heterogeneity of macrophages between the young group and aging group. Since the similarity of infrared spectral profiles can be visualized by plotting the first two principal components (PC1 and PC2) and the dots distribution in the PCA demonstrated the presence of heterogeneity among population. In order to distinguish the subtle cellular biochemical phenotypic heterogeneity, we conducted PCA based on the corresponding second derivative spectra. As our expectation, these analysis results likewise to indicate the biochemical phenotypic heterogeneity of detected single macrophages were small and the control macrophages shared a satisfactory clustering and similarity (Fig. 2C). On the contrary, the PCA results of Inter-group macrophages demonstrated the presence of significant biochemical phenotypic heterogeneity (Fig. 2D).

As shown in Fig. 2D, one sample out of 40 senescent macrophage populations was excluded from 95% confidence ellipse, oval marked by red dotted line, while 40 non-senescent macrophage populations were all within 95% confidence ellipse, oval marked by black dotted line. It was converted into Table 2.

**Table 2:** Macrophage senescence was identified by SR-FTIR and SA-β-gal staining

SR-FTIR microspectroscopy scanning	SA-β-gal staining	
	Senescent macrophage	Non-senescent macrophage
Senescent macrophage	39	1
Non-senescent macrophage	0	40

The estimated sensitivity of SR-FTIR microspectroscopy scanning to detect or determine senescence of alveolar macrophages was 39/(39 + 0) × 100% = 100% and the corresponding estimated specificity was 40/(40 + 1) × 100% = 97.6%. Perhaps we need to expand the experimental sample size or further experiments to obtain more accurate sensitivity and specificity of this technique (Fig. 2E and F). The corresponding loading plots of PC1 and PC2 of Fig. 2D provided information about the spectral features which give rise to the heterogeneity. According to previous studies, the cell-to-cell Euclidean distance could reflect the similarity and difference among cells population for evaluating the phenotypic heterogeneity, we furtherly employed cell-to-cell Euclidean distance quantitative analysis from the second derivative spectra to distinguish the cellular biochemical phenotypic heterogeneity. Similar to PCA, frequency distribution histograms of cell-to-cell Euclidean distances (Fig. 2G) exhibited the differences between two groups had taken place after aging and these differences could be detected by synchrotron FTIR micro spectroscopy. But the difference of Inter-group macrophages shown by hierarchical clustering (HAC) heatmap (Fig. 2H) was not obvious. In addition, we comparative analyzed the fluctuations of biochemical molecular functional groups between the two macrophage populations (Table S1) and semi-quantitative comparative statistic of specific functional groups (Fig. S1).

Owing to infrared spectra that contain various information of chemical components, like lipids, proteins, and nucleic acid and carbohydrates. With the goal of finding out what biochemical components were involved in contributing biochemical phenotypic heterogeneity transition among macrophage during the process of aging and evaluating the role of these biochemical components played in promoting the biochemical phenotypic heterogeneity, we conducted a series of PCA and cell-to-cell Euclidean distance analysis comparisons between In-group (control macrophages) and Inter-group (control macrophages and senescent macrophages).

### Analyzing the biochemical phenotype and phenotypic heterogeneity in lipids region (3000–2800 cm^−1^ and 1480–1300 cm^−1^)

**Fig. 3:**
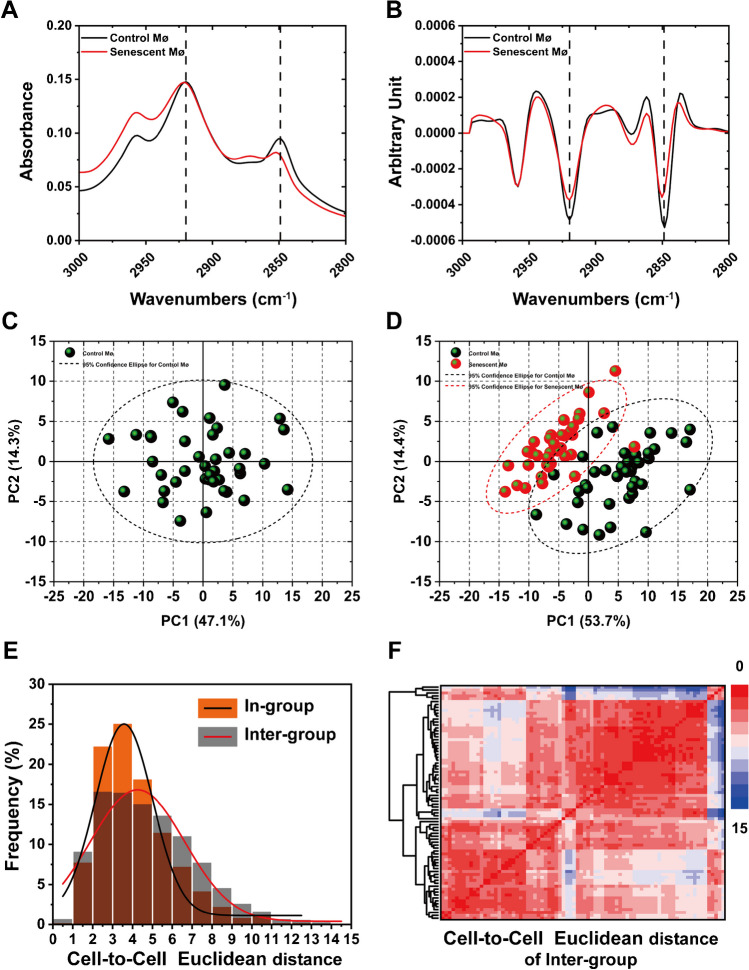
Quantitative analysis macrophage phenotype and phenotypic heterogeneity based on lipids spectrum region. **(A** and **B** The average spectra and second derivative spectra of control macrophages and senescent macrophages. **C** PCA of in-group (control macrophage only), **D** PCA of Inter-group (control macrophages and senescent macrophages), the black dots represent samples of control macrophages and red dots represent samples of senescent macrophages; the ellipse circled by the dotted lines are the 95% confidence ellipse; control (black) and senescent (red). **E** Frequency distribution histograms of cell-to-cell Euclidean distances; Inter-group: control macrophages and senescent macrophages. **F** Hierarchical clustering (HAC) heatmap of cell-to-cell Euclidean distances

Similarly, we calculated the average spectra (Fig. 3A and Fig. S2A) and corresponding second derivative spectra in the lipid region (Fig. 3B and Fig. S2B) which reflected the fluctuation of absorption peaks between the two groups. The clustering situation of control macrophages (black dots) and senescent macrophages (red dots) were visualized in the PCA images (Fig. 3C and D and Fig. S2C and Fig. S2D) by plotting the first two principal components (PC1 and PC2). The PCA of Inter-group demonstrated the phenotypic heterogeneity among the two macrophages population at single cell level (Fig. 3D). Frequency distribution histograms comparison (Fig. 3E and Fig. S2E) and hierarchical clustering (HAC) heatmap (Fig. 3F and Fig. S2F) show that the cell-to-cell Euclidean distances between the two macrophage populations were longer than they were in In-group, which indicates intercellular heterogeneity of macrophages increased during aging. The corresponding loading plots of PC1 and PC2 of Fig. 3D and Fig. S2D are respectively shown in Fig. S4A and Fig. S4C. All above results suggested senescent macrophages exhibited phenotypic heterogeneity of lipids during senescence.

### Analyzing the biochemical phenotype and phenotypic heterogeneity in proteins region during macrophage senescence (1800–1480 cm^−1^)

**Fig. 4:**
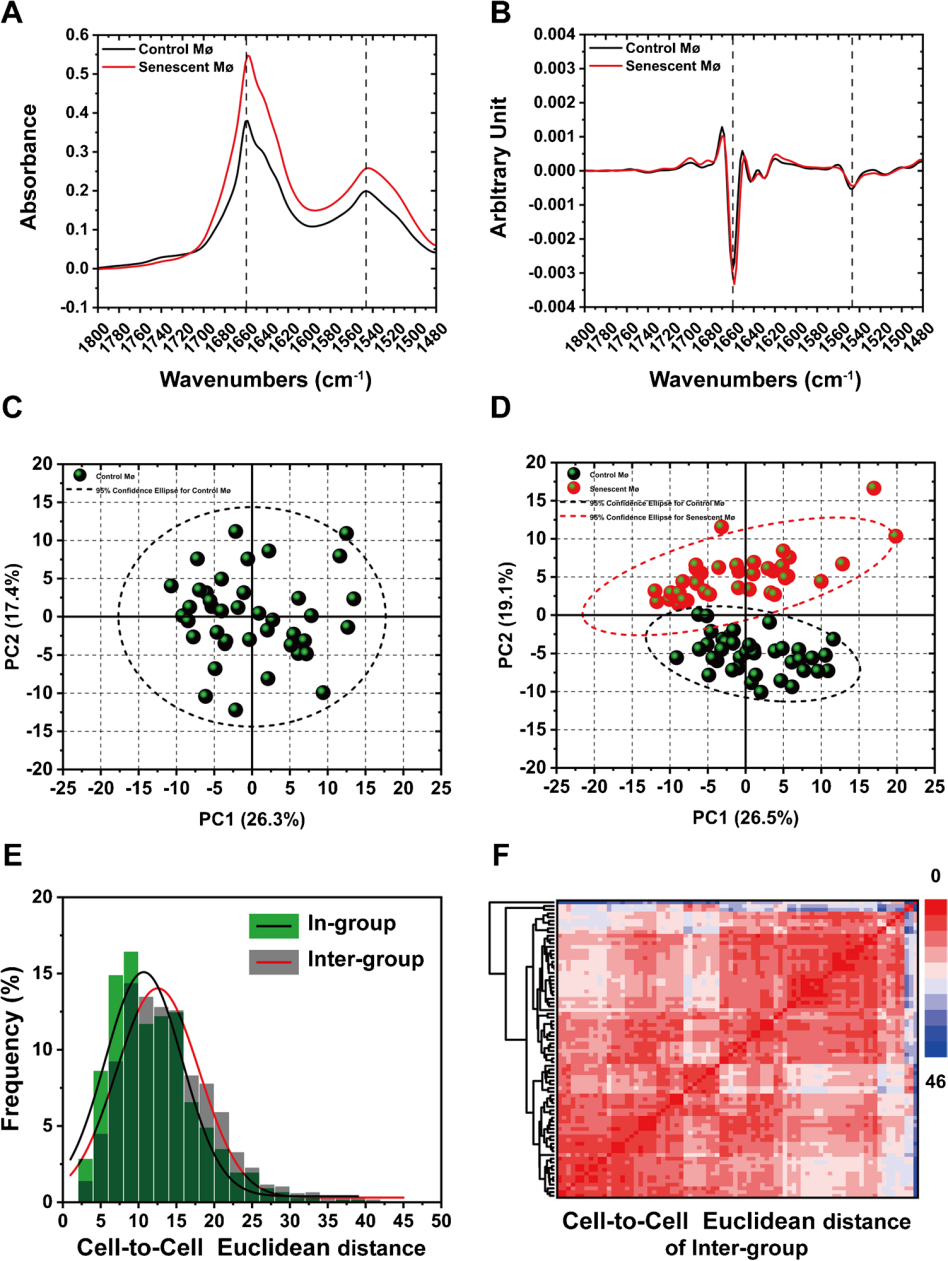
Quantitative analysis macrophage phenotype and phenotypic heterogeneity in proteins region. **A** and **B** The average and second derivative spectra of control and senescent macrophages. **C** PCA of In-group (control macrophages). **D** PCA of Inter-group (control macrophages and senescent macrophages), the black dots represent samples of control macrophages and red dots represent samples of senescent macrophages; the ellipse circled by the dotted lines are the 95% confidence ellipse; control (black) and senescent (red). **E** Frequency distribution histograms of cell-to-cell Euclidean distances; Inter-group: control macrophages and senescent macrophages. **F** Hierarchical clustering (HAC) heatmap of cell-to-cell Euclidean distances

We also calculated the average spectrum (Fig. 4A) and corresponding second derivative spectrum of macrophages in the proteins area (Fig. 4B). The average spectrogram shows the fluctuation of absorption peaks between the two cell populations. The PCA of Inter-group demonstrated the proteins phenotypic heterogeneity among the two macrophages population (Fig. 4C and D). Frequency distribution histograms comparison (Fig. 4E) and hierarchical clustering (HAC) heatmap (Fig. 4F) of Inter-group indicated that cell-to-cell Euclidean distances between the two macrophage populations were short. The corresponding loading plots of PC1 and PC2 of Fig. 4D is shown in Fig. S4B. These results suggested macrophages also exhibited phenotypic heterogeneity of proteins during the senescence progress.

## Conclusions

To sum up, we took use of a label free phenotypic characterizing method by combining SR-FTIR microspectroscopy technology system and statistical calculating the similarities and differences among single senescent macrophage infrared spectra.

Through this SR-FTIR microspectroscopy and heterogeneity analysis, we obtained exact evidence of changes in senescent macrophages at the single cell level. All these results are suggestive of the main biological and chemical changes after macrophage senescence occurred in proteins, cholesterol ester, and fatty acids. Meanwhile, we demonstrated the biochemical phenotype and phenotypic heterogeneity in senescent macrophages relatively comprehensively.

Although PCA analysis was executed well, with the application of computing method improvement, machine learning, or deep learning may replace PCA in the future. We also plan to introduce and apply deep learning in our future research. Moreover, we found biochemical phenotype and phenotypic heterogeneity during macrophage senescence through analyzing the similarity between the comprehensive biochemical molecular substances spectra. Biochemical phenotype and phenotypic heterogeneity are important and valuable for studying the senescent macrophage phenotypic conversion and potential function biochemical metabolism in pathophysiology progress. All these highlight the potential of using SR-FTIR microspectroscopy to provide alternative biochemical information with significant advantages beyond traditional methods. Although the technique does not necessarily provide individual molecular information, it has considerable advantages in providing complete biochemical macromolecular information of the single-cell sample. For example, if biochemical phenotypic differences in macrophages with different genotypes or treated with different interventions could be detected and grouped by SR-FTIR microspectroscopy after senescence, it would be reasonable to assume that the genes or treatments affected the metabolism related to macrophage senescence. It is of great significance to provide an evaluation method or clue for the study cell functions related to cellular material metabolism of macrophage senescence. In other words, it may have the potential to develop into a new method to evaluate the anti-senescence phenotypic transformation therapies targeting biochemical metabolism and to provide insights on new therapies beneficial for senescence.

### Supplementary information

ESM 1 (DOCX 1570 kb)

## Data Availability

The data supporting the findings of this study without undue reservation and will be available from the corresponding authors upon reasonable request.
