# Supplementary material for: Quantitative biochemical phenotypic heterogeneity of senescent macrophage at a single cell level by Synchrotron Radiation Fourier Transform Infrared Microspectroscopy
Source: Mikrochim Acta. 2023 Sep 28;190(10):416. doi: 10.1007/s00604-023-05980-z (PMC10539409; doi:10.1007/s00604-023-05980-z)
Supplement: Supplementary file 1 — (DOCX 1570 kb) [file 604_2023_5980_MOESM1_ESM.docx]

**Electronic Supplementary Material**

**Quantitative biochemical phenotypic heterogeneity of senescent macrophage at a single cell level by Synchrotron Radiation Fourier Transform Infrared microspectroscopy**

Xiaolong Sheng^1, 2^, Jie Wu^1, 2^, Xun Wu^1, 2^, Lianghui Gong^1, 2^, Min Su^1, 2^, Jinming Tang^1, 2^, Desong Yang^1, 2 *^, Wenxiang Wang^1, 2 *^

*1. The Second Department of Thoracic Surgery, Hunan Cancer Hospital/the Affiliated Cancer Hospital of Xiangya School of Medicine, Central South University, Changsha, China.*

*2. Hunan Clinical Medical Research Center of Accurate Diagnosis and Treatment for esophageal carcinoma.*

* Correspondence should be addressed to Desong Yang (**yangdesong@hnca.org.cn**) and Wenxiang Wang ([**wangwenxiang@hnca.org.cn**](mailto:wangwenxiang@hnca.org.cn)).

**Statistical analysis for Table S1 and Figure S1**

All statistical analyses were performed with the SPSS software (version 26.0; SPSS, IBM Inc, New York, USA). The data were expressed as the mean ± standard deviation. All data were tested for normality based on the Shapiro-Wilk test first. For comparison between two groups, unpaired two-tailed Student's t-test were used for data subject to normal distribution and Mann-Whitney U test for data subject to non-normal distribution. ***P*** < 0.05 was considered to be statistically significant. All experiments were randomized, and investigators were blinded to experimental progress and outcomes assessment. “ns” meant the difference was not statistically significant, *, *** and**** represented the degree of significance as ***P*** < 0.05, ***P*** < 0.01 and ***P*** < 0.0001, respectively.

**Table S1** SR-FTIR spectral absorption bands comparison of Inter-group macrophages

| Functional group | Absorption Wavenumber (cm−1) | |
| --- | --- | --- |
|  | Control macrophage | Senescent macrophages |
| **ν_as_** (C-H_2_) | 2919.86 + 1.53 | 2922.32 +6.08 * |
| **δ** (C-H_2_) | 1458.17 + 4.24 | 1450.52 + 4.70 **** |
| **ν_s_** (C=O) of COO^-^ | 1398.03 + 2.70 | 1397.44 + 4.29 ns |
| **ν_as_** (P=O) of PO^2-^ | 1237.56 + 2.31 | 1239.80 + 2.37 **** |
| **ν_s_**（C-OH、C-O-C、C-O-P） | 1080.08 + 5.57 | 1084.27 + 5.24 *** |

**ν_s_/ ν_as_:** symmetric/asymmetric stretching vibration; **δ:** scissoring vibration.

**Table S1.** Corresponding fluctuations of the functional groups of macrophage. The values are the Mean ± SD for each group. Comparisons were done by Student’s t test. *, *** and**** represented the degree of significance as **P** < 0.05, **P** < 0.01 and **P** < 0.0001, respectively.

**Figure S1: Specific functional group comparison of control and senescent macrophages**


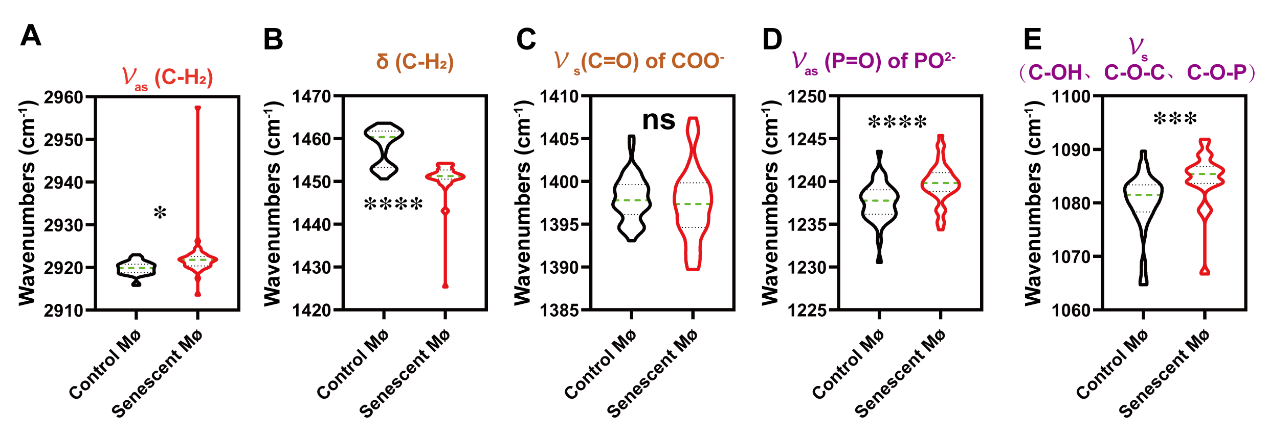


**Fig. S1** Specific functional group comparison of control and senescent macrophages. (A) - (E) display comparison of ν_as_ (C-H_2_) , δ (C-H_2_), νs (C=O) of COO^-^, ν_as_ (P=O) of PO^2-^ and ν_s_（C-OH、C-O-C、C-O-P）respectively. ν_s_/ ν_as_: symmetric/asymmetric stretching vibration; δ: scissoring vibration.

**Figure S2: Analyzing the effect of lipids (1480-1300 cm^−1^) on biochemical phenotypic heterogeneity during macrophage senescence progress**

**
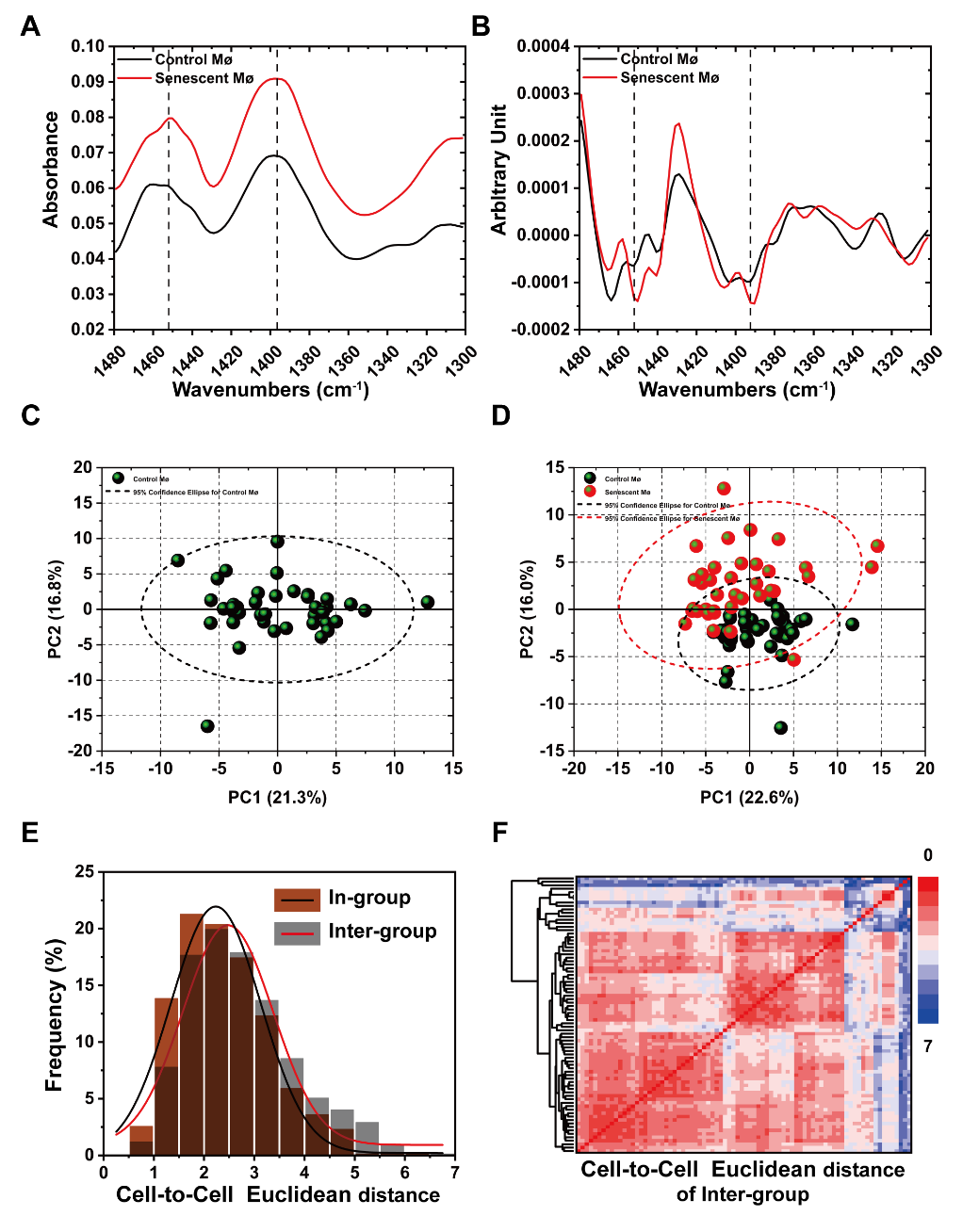
**

**Figure S2.** Quantitative analysis of lipids (in specific spectral wavenumbers 1480-1300 cm^−1^) biochemical phenotypic heterogeneity in macrophage senescence. (A) and (B) The average spectra and second derivative spectra of control macrophages and senescent macrophages. (C) PCA of In-group (control macrophages). (D) PCA of Inter-group (control macrophages and senescent macrophages). (E) Frequency distribution histograms of cell-to-cell Euclidean distances; Inter-group: control macrophages and senescent macrophages. (F) Hierarchical clustering (HAC).

**Figure S3: Analyzing the effect of nucleic acids and carbohydrates on biochemical phenotypic heterogeneity during macrophage senescence progress**

**
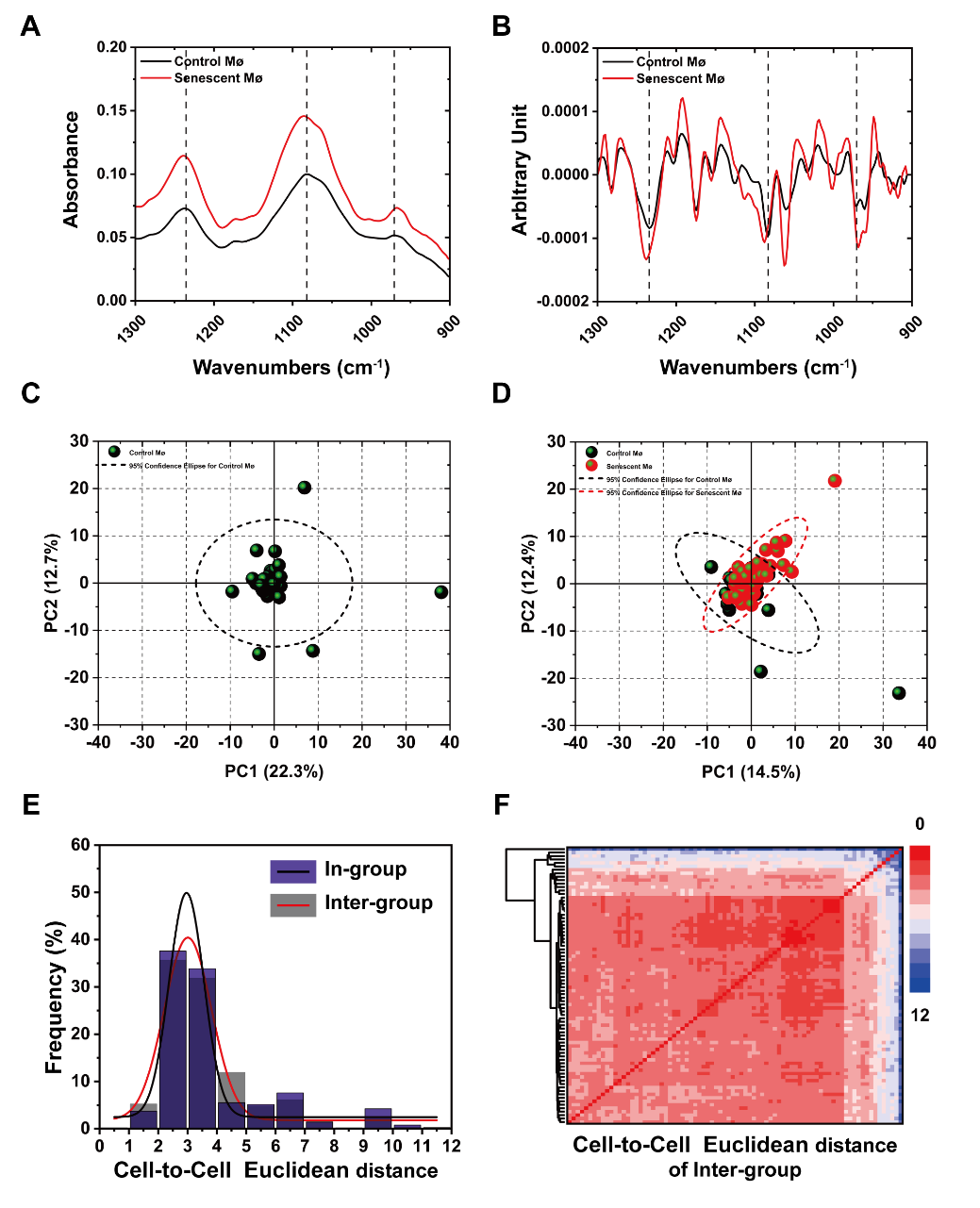
**

**Figure S3.** Quantitative analysis of nucleic acids and carbohydrates heterogeneity in macrophages senescence. (A) and (B) The average spectra and second derivative spectra of control macrophages and senescent macrophages. (C) PCA of In-group (control macrophages). (D) PCA of Inter-group (control macrophages and senescent macrophages). (E) Frequency distribution histograms of cell-to-cell Euclidean distances; Inter-group: control macrophages and senescent macrophages. (F) Hierarchical clustering (HAC).

**Figure S4:** **The corresponding loading plots of PC1 and PC2**

**
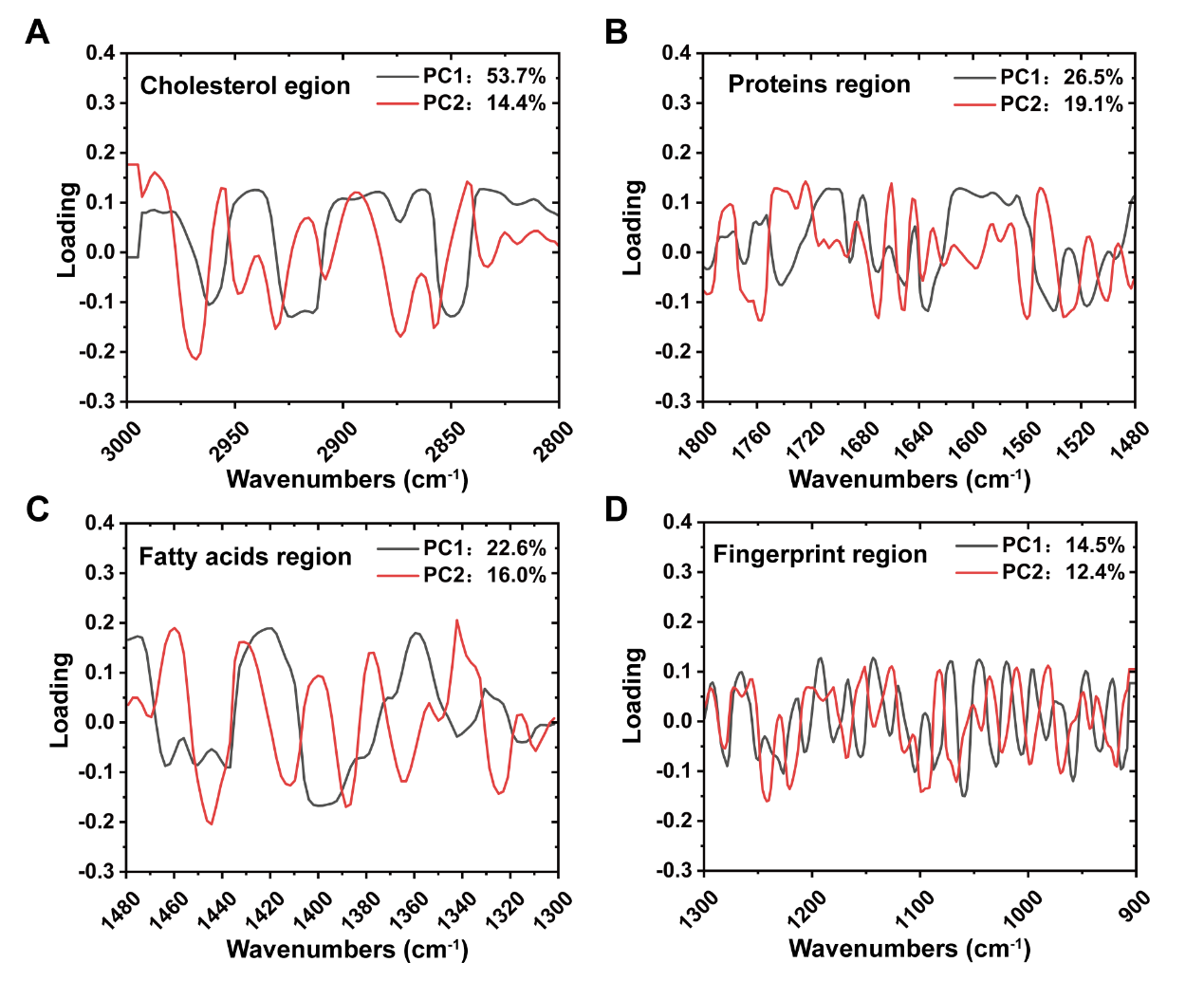
**

**Figure S4**. Loading plots of PC1 and PC2. (A) Loading plots of PC1 and PC2 of Fig. 3D. (B) Loading plots of PC1 and PC2 of Fig. 4D. (C) Loading plots of PC1 and PC2 of Fig. S2D. (D) Loading plots of PC1 and PC2 of Fig. S3D.
